# Modified hindfoot alignment radiological evaluation and application in the assessment of flatfoot

**DOI:** 10.1186/s12891-023-06824-w

**Published:** 2023-08-29

**Authors:** Jing-Qi Liang, Yan Zhang, Liang Liu, Xiao-Dong Wen, Pei-Long Liu, Xin-Quan Yang, Xiao-Jun Liang, Hong-Mou Zhao

**Affiliations:** Foot and Ankle Surgery Department, Honghui Hospital of Xi’an Jiaotong University, Xi’an, China

**Keywords:** Hindfoot alignment, Radiology measurement, Flatfoot

## Abstract

**Background:**

Alignment is indispensable for the foot and ankle function, especially in the hindfoot alignment. In the preoperative planning of patients with varus or valgus deformity, the precise measurement of the hindfoot alignment is important. A new method of photographing and measuring hindfoot alignment based on X-ray was proposed in this study, and it was applied in the assessment of flatfoot.

**Methods:**

This study included 28 patients (40 feet) with flatfeet and 20 volunteers (40 feet) from January to December 2018. The hindfoot alignment shooting stand independently designed by our department was used to take hindfoot alignment X-rays at 10 degree, 15 degree, 20 degree, 25 degree, and 30 degree. We measured the modified tibio-hindfoot angle (THA) at the standard hindfoot aligment position (shooting at 20 degree) and evaluated consistency with the van Dijk method and the modified van Dijk method. In addition, we observed the visibility of the tibiotalar joint space from all imaging data at five projection angles and evaluated the consistency of the modified THA method at different projection angles. The angle of hindfoot valgus of flatfoot patients was measured using the modified THA method.

**Results:**

The mean THA in the standard hindfoot aligment view in normal people was significantly different among the three evaluation methods (P < .001). The results from the modified THA method were significantly larger than those from the Van Dijk method (P < .001) and modified Van Dijk method (P < .001). There was no significant difference between the results of the modified THA method and the weightbearing CT (P = .605), and the intra- and intergroup consistency were the highest in the modified THA group. The tibiotalar space in the normal group was visible in all cases at 10 degree, 15 degree, and 20 degree; visible in some cases at 25 degree; and not visible in all cases at 30 degree. In the flatfoot group, the tibiotalar space was visible in all cases at 10 degree, visible in some cases at 15 degree and 20 degree, and not visible in all cases at 25 degree and 30 degree. In the normal group, the modified THA was 4.84 ± 1.81 degree at 10 degree, 4.96 ± 1.77 degree at 15 degree, and 4.94 ± 2.04 degree at 20 degree. No significant differences were found among the three groups (P = .616). In the flatfoot group, the modified THA of 18 feet, which was visible at 10 degree, 15 degree and 20 degree, was 13.58 ± 3.57 degree at 10 degree, 13.62 ± 3.83 degree at 15 degree and 13.38 ± 4.06 degree at 20 degree. There were no significant differences among the three groups (P = .425).

**Conclusions:**

The modified THA evaluation method is simple to use and has high inter- and intragroup consistency. It can be used to evaluate hindfoot alignment. For patients with flatfeet, the 10 degree position view and modified THA measurement can be used to evaluate hindfoot valgus.

## Introduction

Patients with congenital deformities of the foot always have varus or valgus of the hindfoot, joint degeneration, including traumatic ankle instability and ankle arthritis, are also associated with abnormalities in hindfoot alignment [1, 2]. The key to formulating a reasonable therapeutic regimen is to accurately evaluate the alignment of the hindfoot on the coronary surface [3–5]. Proposed a radiological measurement and evaluation method associated with hindfoot alignment in 1995 by Saltzman [6], but the author only divided hindfoot deformities into varus and valgus through the location where axis extension of tibia is located at the lowest point of the calcaneus. Recently, some modified measurement methods regarding the hindfoor view have mainly focused on the definition of the axis of the calcaneus [7]. However, previous measurement methods were to compare the included angle between the axis of the tibia and that of the calcaneus, while varus or valgus subtalar joints and varus or valgus talus possibly exist between the calcaneus and tibia. Therefore, the previous measurement methods fail to adequately reflect the real situation regarding varus or valgus of the hindfoot. Burssens found a larger difference between the measured results of hindfoot alignment with 2D CT and 3D CT [8], which were compared through weightbearing CT. Weightbearing CT, which can provide precise anatomical information, can serve as the “gold standard” to measure hindfoot alignment. However, it is necessary to purchase special weightbearing CT equipment for the foot and ankle parts, and the measurement method requires professional imaging software. Therefore, weightbearing CT has very limited clinical applications and has been difficult to popularize.

Accordingly, regarding the relationship between the tibia and hindfoot, namely, the relationship between the tibia and subtalar complex, it is still necessary to find for an X-ray measurement method of hindfoot alignment with accuracy, simplicity, efficiency and high consistency. Therefore, X-rays from the hindfoot alignment location need to clearly display the talus dome joint surface. Traditional photographic methods can display the talus dome joint surface in normal persons but frequently fail to display the talus dome joint surface in patients with flatfoot or talipes cavus due to changes in the arch; therefore, hindfoot alignment fails to be accurately evaluated. The photographic methods were modified in the present study. A new method to measure hindfoot alignment was proposed based on X-ray scans, and the reliability of the measurement method and its application to patients with flatfoot were assessed as follows. The research was approved by the ethics committee of our hospital.

## Materials and methods

### General materials

Twenty-eight patients (40 feet) with flatfoot deformity diagnosed in our hospital from January 2018 to December 2020 were included in this research, including 17 males and 11 females with an average age was 36.4 ± 9.6 years and an average BMI of 27.4 ± 3.6 kg/m^2^. There were 20 volunteers (40 feet), including 12 males and 8 females with an average age was 33.5 ± 6.7 years and an average BMI of 25.3 ± 3.9 kg/m^2^. There were no significant differences in age, sex, side or BMI between groups (Table 1). All the patients and volunteers underwent photography of hindfoot alignment on the hindfoot alignment photography frame autonomously designed by our department, as well as X-ray scans of adem position with weightbearing on the foot. All imaging data were used in the subsequent measurements.

**Table 1:** Data associated with patients in the flatfoot group and volunteer group

	Flatfoot group (n = 28)	Volunteer group (n = 28)	P value
Cases	40	40	-
Age	36.4 ± 9.6	33.5 ± 6.7	0.251
Sex (male/female)	17/11	12/7	0.866
Side (left/right)	22/18	20/20	0.654
BMI (kg/m^2^)	27.4 ± 3.6	25.3 ± 3.9	0.061

BMI, body mass index

### Inclusion and exclusion criteria

The inclusion criteria for those with flatfoot deformity were as follows: ① adult flatfoot deformity with hind valgus and interior longitudinal arch declination; ② Meary’s angle on the X-ray in the lateral position of the weightbearing foot < 5 degree; ③ lateral or bilateral flatfoot deformity; and ④ voluntary participation and all the imaging data available. The inclusion criteria for volunteers were as follows: ① normal adult volunteer without deformity for feet clinically confirmed by physical examination; and ② Meary’s angle on the X-ray in the lateral position of the weightbearing foot less than ± 4 degree.

Both groups use the same exclusion criteria. The exclusion criteria were as follows: ① ankle with relevant trauma or surgical history; ② ankle joint degeneration diseases including osteoarthritis, rheumatoid arthritis, Charcot osteoarthropathy, or gout arthritis; ③ consciousness and cognitive disorder or serious mental disease and failure to cooperate during the examination; and ④ preparing for pregnancy or pregnancy.

### Design of the photography frame and photographic method for hindfoot alignment

**Fig. 1:**
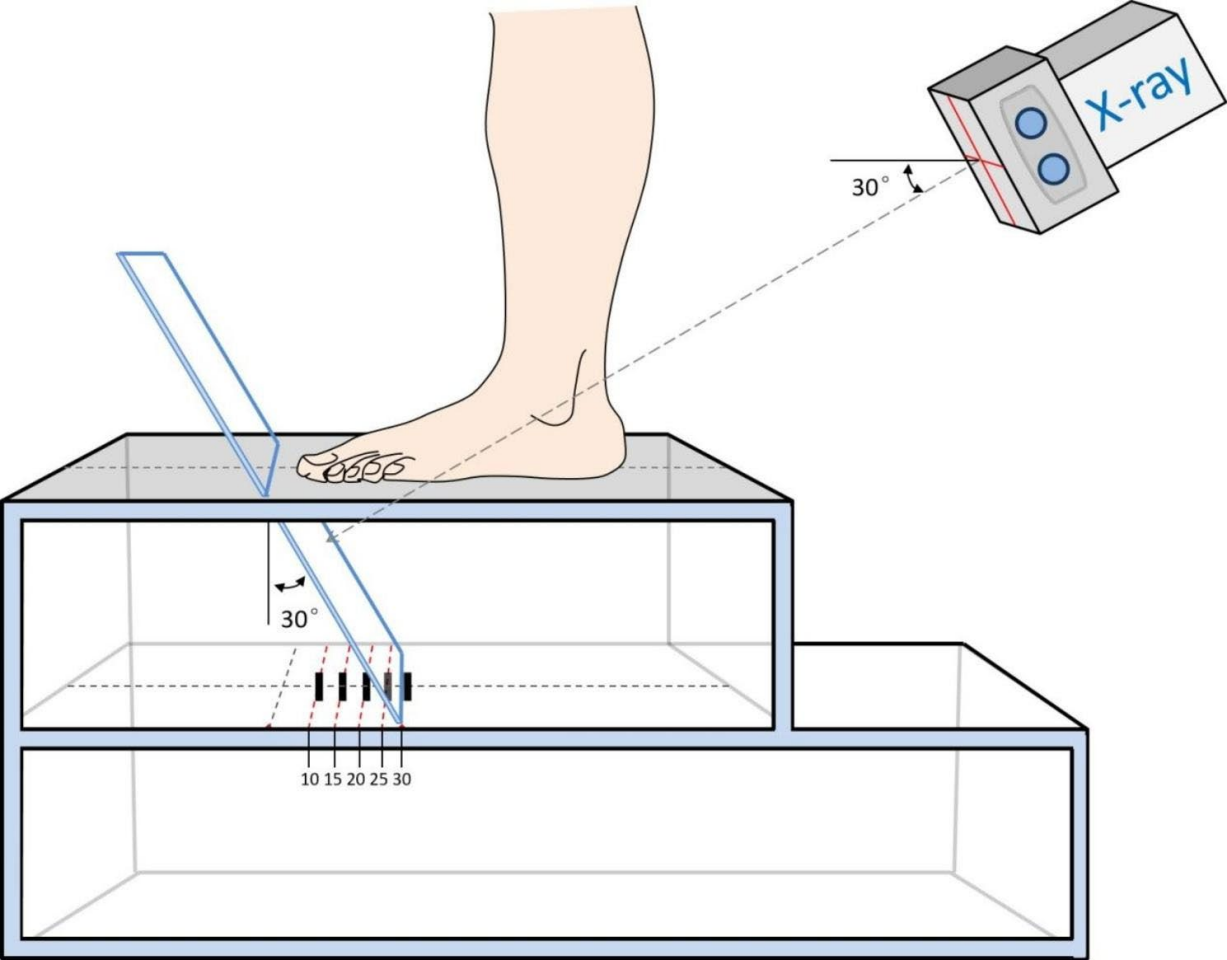
Modified photographic method for hindfoot alignment location

A small card slot was established, and a removeable board was designed for placement on the first baffle based on the premeasured angle position through a photographic frame for hindfoot alignment that was autonomously designed by our department and corresponded to one of five insertion angles of the X-ray imaging board (10 degree, 15 degree, 20 degree, 25 degree and 30 degree); the insertion port of the X-ray imaging board was in the center of the second standing board. Patients stood on the second board when being photographed, with feet parallel to each other and appropriately aligned to the imaging board. The X-ray bulb tube was appropriately aligned to the midpoint of the bilateral ankle joint ligature and vertical to the imaging board (Fig. 1). All the included patients and volunteers were photographed with five groups of X-ray scans of hindfoot alignment location at 10 degree- 30 degree.

### Method to measure hindfoot alignment position

**Fig. 2:**
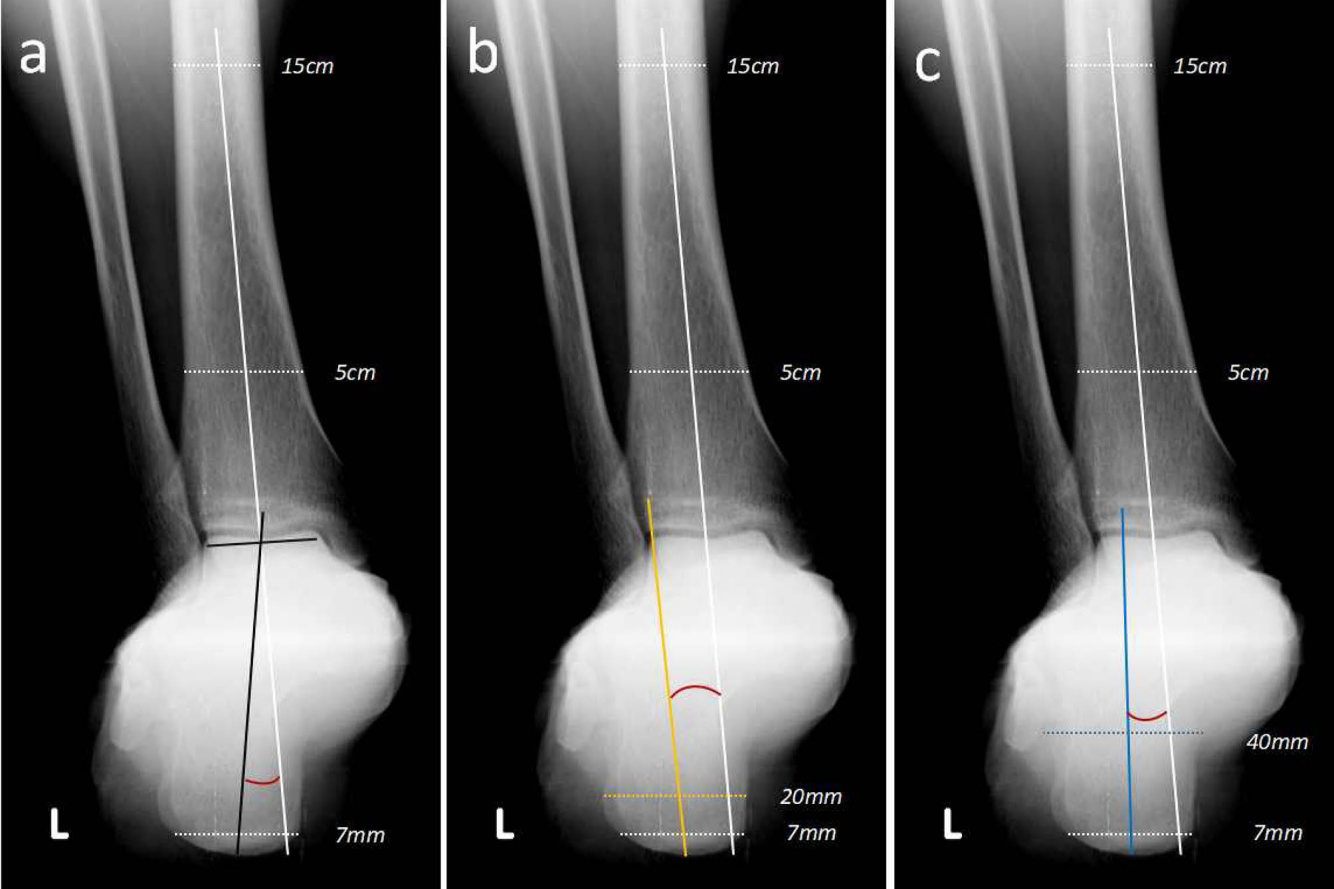
Three methods to measure hindfoot alignment. **a** Modified method to measure tibio-hindfoot angle and the included angle between the axis of the tibia and that of the hindfoot. **b** Van Dijk method to measure hindfoot alignment and the included angle between the axis of the tibia and that of the calcaneus (the ligature of the midpoints were 7 and 20 mm above the lowest point of the calcaneus). **c** Modified van Dijk method to measure hindfoot alignment and the included angle between the axis of tibia and that of the calcaneus (the ligature of the midpoints were 7 and 40 mm above the lowest point of the calcaneus)

The modified measurement method of the tibia-hindfoot angle (THA) was used, with the following specific measures: ① tibia axis: the ligature of the midpoints of the bilateral cortical bone of the tibia at 5 and 15 cm above the distal end joint surface of the tibia; ② hind axis: the ligature of the midpoint of the talus dome joint and the midpoint of the ligature of the bilateral cortical bone at the place more than 7 mm above the lowest point of the calcaneus. The included angle between the tibia axis and hind axis was measured as the modified THA (Fig. 2A).

### Control measures using the traditional method with three methods to measure hindfoot alignment

Standard traditional method X-ray scans from 20 volunteers with 40 feet, namely, X-ray hindfoot alignment positions at the 20 degree projection position, were taken as the research object. The angle of hindfoot alignment was measured by the modified THA measurement method, the van Dijk method, and the modified van Dijk method (Fig. 2). The between-group consistency and intragroup consistency for the three measurement methods were evaluated. The differences among the three measured results were compared, which were also compared with the THA results using weightbearing CT reported by Burssens [8].

### Visibility of tibiotalar joint space under different photographic angles

**Fig. 3:**
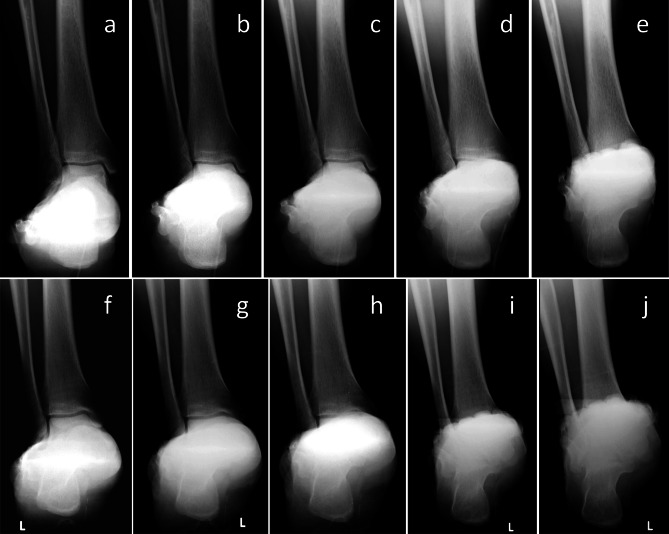
**a-e** X-ray scans of the hindfoot alignment position of normal volunteers at 10 degree, 15 degree, 20 degree, 25 degree and 30 degree. **f-j** X-ray scans of the hindfoot alignment position of patients with flatfoot at 10 degree, 15 degree, 20 degree, 25 degree and 30 degree

X-ray scans for hindfoot alignment using five projection angles for the patients with flatfoot and normal volunteers were included, and an evaluation and statistical analysis of visibility of the tibiotalar joint was performed; that is, when all talus domes were clear and visible, this was defined as the tibiotalar joint space being visible (Fig. 3).

### Consistency of modified THA under different projection angles

X-ray scans associated with all the visible tibiotalar joint spaces of the patients with flatfoot and normal volunteers were measured by the modified THA measurement method, and the consistency of THA in the flatfoot patient group and normal volunteer group under different projection angles was evaluated.

### Statistical analysis

Statistical analysis was performed with SPSS version 18.0 (SPSS Inc., Chicago, IL). Between-group and intragroup consistency was analyzed through the intraclass correlation coefficient (ICC) [9]. These correlations were categorized as follows: ICC < 0.4, bad; 0.4 < ICC < 0.59, qualified; 0.6 < ICC < 0.74, good; and ICC > 0.74, excellent. A P value less than 0.05 was considered to be a significant difference. All results are displayed as the mean ± standard deviation.

## Results

### Control research with three methods to measure hindfoot alignment

The angles of hindfoot alignment by three measurement methods and between-group and intragroup ICC results are shown in Table 2. The angles of hindfoot alignment by the three measurement methods were significantly different (P < .001). Further between-group comparisons showed that the result measured by modified THA was significantly greater than that measured by the van Dijk method (P < .001) and modified van Dijk method (P < .001). The results of the three groups were compared with the result (THA = 4.60 ± 3.70) of hindfoot alignment for normal persons measured by weightbearing CT. There was no significant difference between the result measured by the modified THA put forward by the present research and the result measured by weightbearing CT (P = .605), and the results obtained using the van Dijk method (P = .006) and modified van Dijk method (P = .038) were significantly lower than the result measured by modified CT. Based on these results, between-group ICC and intragroup ICC with the three measurement methods were excellent, and the consistency of the modified THA measurement method proposed in this research was the best.

**Table 2:** Evaluation of the results of hindfoot alignment by different methods for the standard Saltzman location (n = 40)

	Mean	SD	Intergroup ICC	Intragroup ICC
Modified tibio-hindfoot angle	4.94	2.05	0.941	0.928
Van Dijk method	2.90	1.03	0.798	0.776
Modified van Dijk method	3.29	1.48	0.834	0.855

ICC, intraclass correlation coefficient

### Visibility of the tibiotalar joint under different photographic angles

**Fig. 4:**
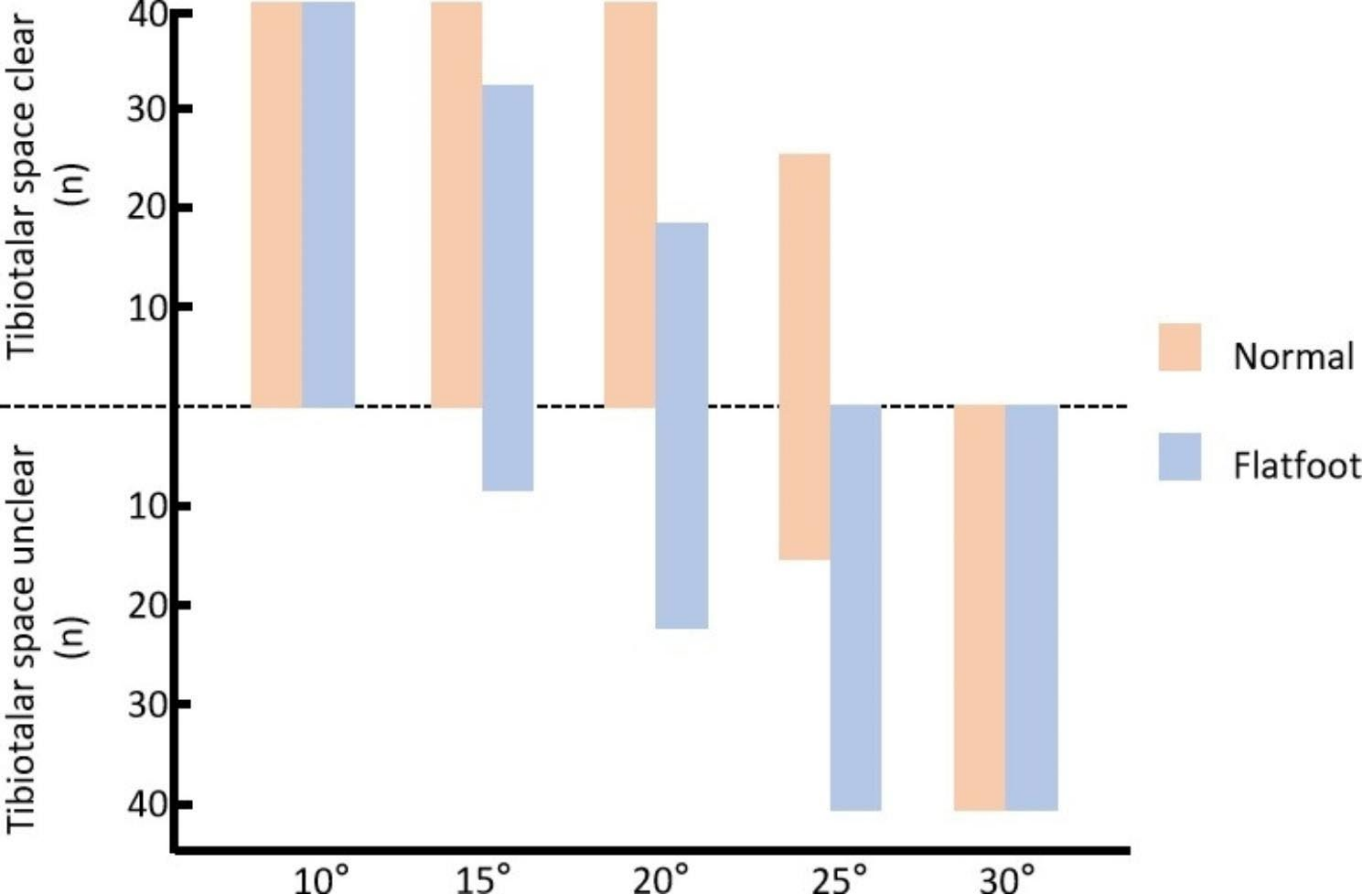
Visibility of the tibiotalar joint across the five projection angles for the flatfoot group and normal group

The visibility of the tibiotalar joint in the two groups of subjects was as follows: flatfoot group: all were visible at 10 degree, 32 feet could be seen at 15 degree, 18 feet could be seen at 20 degree, and none of them could be seen at 25 degree and 30 degree; normal volunteer group: all of them could be seen at 10 degree, 15 degree and 20 degree, 28 feet could be seen at 25 degree, and none of them could be seen at 30 degree (Fig. 4).

### Consistency of the modified THA based on different projection angles

Visible imaging data of the tibiotalar joint space for the flatfoot group and normal group at 10 degree, 15 degree and 20 degree were extracted from the five hindfoot alignment results and measured by the modified THA method (Table 3). In the volunteer group, THA at 10 degree was 4.84 ± 1.81 degree, THA at 15 degree was 4.96 ± 1.77 degree, and THA at 20 degree was 4.94 ± 2.04 degree. There were no significant differences across the results from these three angles (P = .616). Tibiotalar joints of 18 patients in the flatfoot group were visible at 10 degree, 15 degree and 20 degree. For these 18 patients, THA at 10 degree was 13.58 ± 3.57 degree, THA at 15 degree was 13.62 ± 3.83 degree, and THA at 20 degree was 13.38 ± 4.06 degree. There were no significant differences among the results from these three angles (P = .425).

**Table 3:** Modified THA values based on different projection angles

	Mean	SD	P-value
Normal 10° (n = 40)	5.24	1.82	0.616
Normal 15° (n = 40)	5.11	1.77	
Normal 20° (n = 40)	4.94	2.05	
Flatfoot 10° (n = 40)	13.41	3.64	0.425*
Flatfoot 15° (n = 40)	12.52	2.94	
Flatfoot 20° (n = 40)	10.38	1.29	

* Refers to the comparison of results from visible tibiotalar joints for 18 feet at 10°, 15° and 20°

## Discussion

**Fig. 5:**
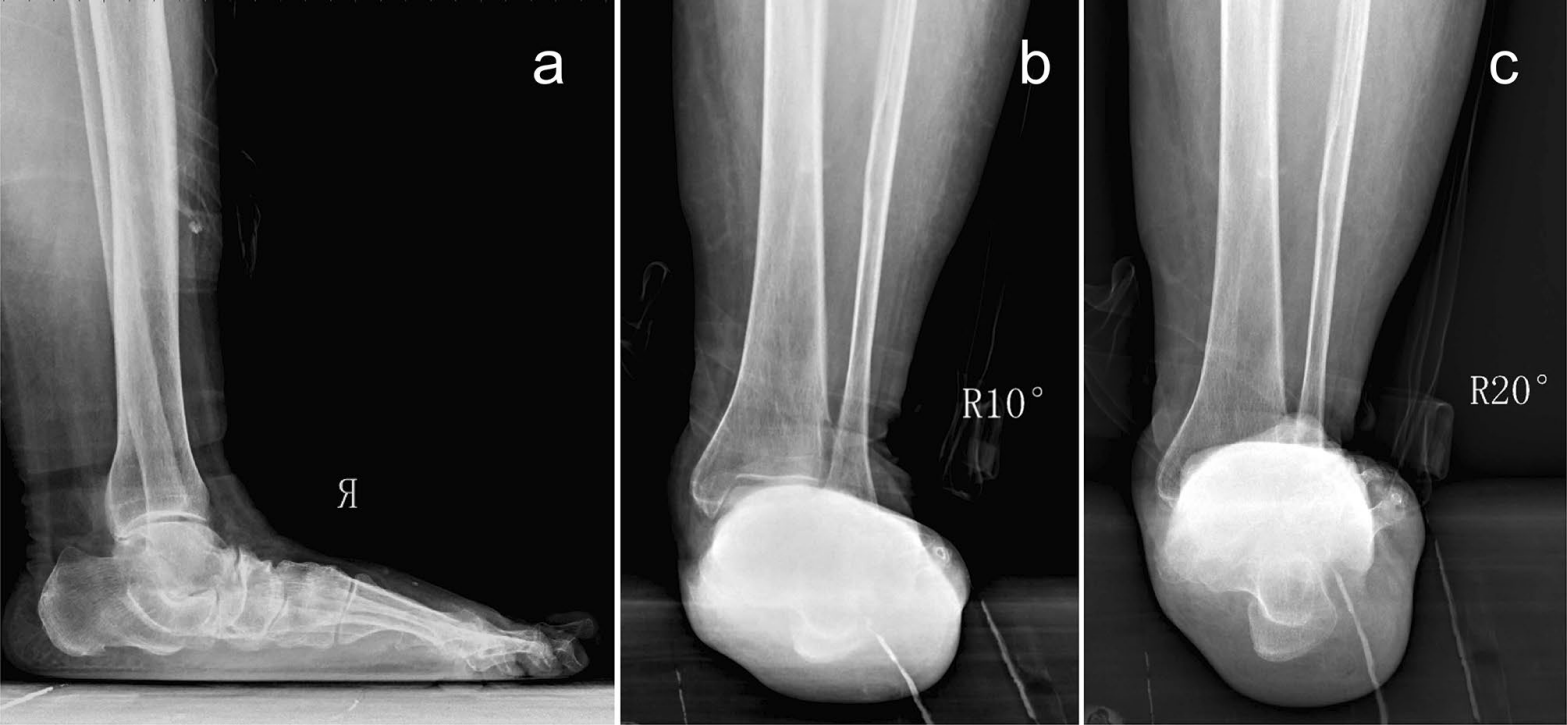
56-year-old female, adult-acquisition flatfoot. **a** The foot longitudinal arch collapse. **b** Tibiotalar joint space can be seen using 10 degree hindfoot alignment photography. **c** The tibiotalar joint failed to be observed using traditional method (20 degree) hindfoot view photography

The ankle joint and tibiotalar joint are the core structures that accomplish complicated hindfoot movement [2]. Varus and valgus tibiotalar joints frequently result in abnormalities of hindfoot alignment, causing changes in weightbearing position, which will cause clinical symptoms such as pain. The evaluation of hindfoot alignment includes a clinical physical examination and imaging evaluation. However, when clinical physical examinations are performed, serious inconsistencies exists among ankle surgeons with extensive experience, due to large differences between observers [10, 11]. Therefore, accurate evaluation of hindfoot alignment is still assisted by imaging examinations [12]. Many investigations have focused on determining an accurate method to evaluate hindfoot alignment allowing the accurate correction of hindfoot deformity to normal anatomical alignment through surgery. Therefore, there is a significant correlation between accurate imaging evaluation and clinical results [13, 14]. Identifying X-ray photography and measurement methods that truly reflect the hindfoot alignment situation can result in good evaluation regarding the degree of deformity. Many anatomical signs and angles reported in many studies accurately display the relationship between the axis of the tibia and the axis of the calcaneus, as well as real hindfoot alignment [5, 6, 15–18]. However, imaging evaluation related to hindfoot alignment is very complicated. It has been reported that the 2D mode of X-ray scans limits the accuracy of hindfoot alignment measurements, and accurate evaluation of hindfoot alignment is hindered by the complicated anatomy of the tibiotalar joint [19, 20]. In all the current methods to measure hindfoot alignment by X-ray scans, the extension of the ligature from two selected points above the tibiotalar joint surface is selected as the axis of the tibia. However, for patients with abnormal hindfoot alignment, such as patients with flatfoot, the traditional hindfoot view fails to photograph the ankle joint in some patients due to bone overlap, which limits the accuracy of measurement of the tibia axis (Fig. 5). Additionally, the hindfoot is traditionally defined as the complex of the talus and calcaneus, so evaluation of hindfoot alignment in traditional methods is supposed to simultaneously consider the possible influence of the accuracy of the varus or valgus subtalar joint and varus or valgus talus.

Another divergence originates from the lack of a standardized measurement method for accurate evaluation in the evaluation of hindfoot alignment [12]. Reilingh [5] reported that different reference points and measurement methods have been used for the evaluation of hindfoot alignment, and the long axis position of the calcaneus was more reliable. Dagneaux [7] compared many modified measurement methods and proposed that the modified van Dijk method should be used for measuring real hindfoot alignment. However, the tibiotalar joint is difficult to observe using various previously available methods for patients with abnormal hindfoot alignment, and it is very hard to use the same bone sign for alignment measurement making it difficult to evaluate. Although various modified photographic and measurement methods of hindfoot alignment have been extensively described, especially for evaluations based on X-ray scans, there are large divergences in clinical applications. Most patients with abnormal hindfoot alignment are hard to evaluate by the current reported methods [10, 19]. Larger measurement errors were caused by inconsistent photography angles or errors in localization when relevant imaging materials were measured [21, 22], which causes difficulty in clinical application. Regarding the modified photographic method and measurement method of hindfoot alignment proposed on the present research, the anatomical location of the new measurement method is clear and stable, and the drawing lines for the tibia axis and hindfoot axis is simple and reliable, with high repeatability. Therefore, the between-group and intragroup consistency of the measured results with the new measurement method were higher than those for the existing measurement methods. CT and 3D reconstruction can clearly reveal the detailed bone structure of the ankle and hindfoot but only provide the anatomical structure of the skeleton [1, 23, 24]. In the non-weightbearing situation, the relationship between the ankle and hindfoot bone joint is completely different from that in the normal weightbearing situation. Therefore, the evaluation of hindfoot alignment while weightbearing is crucial for patients with hindfoot deformities.^24^ Burssens^8^ measured hindfoot alignment by weightbearing CT and proposed a method to measure hindfoot alignment by weightbearing CT. The method can be used to accurately evaluate the relationship between the anatomical form and skeleton of the hindfoot in weightbearing human, so it can serve as the “Gold Standard” for evaluating hindfoot alignment. However, this technology is currently hard to carry out on a large scale because weightbearing CT machines are very expensive with limited application ranges, and accurate measurements are assisted by digital orthopedics software. Therefore, X-ray scans are the most extensively used imaging examination method. Based on the measurement method proposed in the present research, the results of normal persons measured by the modified THA and the results measured by Burssens [8] through weightbearing CT were compared, and no significant differences were found. Compared with expensive photography equipment, as well as the complicated measurement method, the modified THA photography and measurement method proposed by the research is simpler and more practical and easily promoted and applied clinically.

However, this research has some limitations. For example, the real angle of hindfoot alignment of the patients included in this research is unknown without weightbearing CT, and the research results were only compared with the weightbearing CT results reported in other studies, which could possibly influence the accuracy of results. However, the angles of hindfoot alignment evaluated by weightbearing CT reported by Burssens [8] were obtained from normal persons. In this research, evaluations were performed through clinical examinations and X-ray scans of the adem position of the weightbearing foot and the ankle joint in the normal control group to eliminate the existence of abnormal ankle alignment. Therefore, comparability exists among the included persons. Additionally, the research only evaluated the application of the modified THA measurement method to patients with flatfoot, without evaluating patients with cavus/varus foot. Accordingly, these populations can be evaluated in future studies.

## Conclusions

Accurately evaluating hindfoot alignment is very important for surgery preparation for patients with abnormal hindfoot alignment in the clinic. Some disadvantages of measurement and evaluation methods based on X-ray scans exist, with difficulty in evaluating patients with serious hindfoot deformities. Consequently, a modified method was used to measure hindfoot alignment. The modified THA measurement is simple to perform, with higher between-group and intragroup consistency, and it can be used to accurately evaluate hindfoot alignment. For patients with flatfoot, the modified THA measurement method with photography from the 10 degree location can be used to accurately evaluate the degree of hindfoot valgus.

## Data Availability

The datasets used and/or analysed during the current study available from the corresponding author on reasonable request.

## References

[1] Van Bergeyk AB, Younger A, Carson B (2002). CT analysis of hindfoot alignment in chronic lateral ankle instability. Foot Ankle Int.

[2] Coughlin MJ, Mann RA, Saltzman CL. (2007) Surgery of the foot and ankle[M], 8th ed. Philadelphia, pp 951–976.

[3] Johnson JE, Lamdan R, Granberry WF, Harris GF, Carrera GF (1999). Hindfoot coronal alignment: a modified radiographic method. Foot Ankle in.

[4] Kleiger B, Mankin HJ (1961). A roentgenographic study of the development of the calcaneus by means of the posterior tangential view. J Bone Joint Surg Am.

[5] Reilingh ML, Beimers L, Tuijthof GJM, Stufkens SAS, Maas M, Van Dijk CN (2010). Measuring hindfoot alignment radiographically: the long axial view is more reliable than the hindfoot alignment view. Skeletal Radiol.

[6] Saltzman CL, El-Khoury GY (1995). The hindfoot alignment view. Foot Ankle Int.

[7] Dagneaux L, Moroney P, Maestro M (2019). Reliability of hindfoot alignment measurements from standard radiographs using the methods of Meary and Saltzman. Foot Ankle Surg.

[8] Burssens A, Peeters J, Peiffer M, Marien R, Lenaerts T, Vandeputte G, Victor J (2018). Reliability and correlation analysis of computed methods to convert conventional 2D radiological hindfoot measurements to a 3D setting using weightbearing CT. Int J Comput Assist Radiol Surg.

[9] Shrout PE, Fleiss JL (1979). Intraclass correlations: uses in assessing rater reliability. Psychol Bull.

[10] Holly J, Haight DL, Dahm J, Smith DA, Krause (2005). Measuring standing hindfoot alignment: reliability of goniometric and visual measurements. Arch Phys Med Rehabil.

[11] Menz HB (1995). Clinical hindfoot measurement: a critical review of the literature. Foot.

[12] Buck FM, Hoffmann A, Mamisch-Saupe N, Espinosa N, Resnick D, Hodler J (2011). Hindfoot alignment measurements: rotation-stability of measurement techniques on hindfoot alignment view and long axial view radiographs. AJR Am J Roentgenol.

[13] Haleem AM, Pavlov H, Bogner E, Sofka C, Deland JT, Ellis SJ (2014). Comparison of deformity with respect to the talus in patients with posterior tibial tendon dysfunction and controls using multiplanar weight-bearing imaging or conventional radiography. J Bone Joint Surg Am.

[14] Grumbine NA, Santoro JP (1990). The tendo Achillis as it relates to rearfoot position. A new classification for evaluation of calcaneal stance position. Clin Podiatr Med Surg.

[15] Keenan AM, Bach TM (2006). Clinicians’ assessment of the hindfoot: a study of reliability. Foot Ankle in.

[16] Arangio G, Rogman A, Reed JF 3rd. Hindfoot alignment valgus moment arm increases in adult flatfoot with Achilles tendon contracture. Foot Ankle Int. 2009;30(11):1078–82. 10.3113/FAI.2009.1078. 10.3113/FAI.2009.1078 19912718

[17] Sutter R, Pfirrmann CW, Espinosa N, Buck FM (2013). Three-dimensional hindfoot alignment measurements based on biplanar radiographs: comparison with standard radiographic measurements. Skeletal Radiol.

[18] Tuijthof GJ, Herder JL, Scholten PE, Dijk V, Pistecky CN (2004). Measuring alignment of the hindfoot. J Biomech Eng.

[19] Coughlin MJ, Kaz A (2009). Correlation of Harris mats, physical exam, pictures, and radiographic measurements in adult flatfoot deformity. Foot Ankle Int.

[20] Cody EA, Williamson ER, Burket JC, Deland JT, Ellis SJ. (2016) Correlation of Talar Anatomy and Subtalar Joint Alignment on Weightbearing Computed Tomography With Radiographic Flatfoot Parameters. Foot Ankle Int 37(8): 874–881. https://doi.org/1177/1071100716646629. 10.1177/1071100716646629 27137795

[21] Hirschmann A, Pfirrmann CW, Klammer G, Espinosa N, Buck FM (2014). Upright cone CT of the hindfoot: comparison of the non-weight-bearing with the upright weight-bearing position. Eur Radiol.

[22] Burssens A, Peeters J, Buedts K, Victor J, Vandeputte G (2016). Measuring hindfoot alignment in weight bearing CT: a novel clinical relevant measurement method. Foot Ankle Surg.

[23] Mendicino RW, Catanzariti AR, Reeves CL, King GL (2005). A systematic approach to evaluation of the rearfoot, ankle, and leg in reconstructive surgery. J Am Podiatr Med Assoc.

[24] Seltzer SE, Weissman BN, Braunstein EM, Adams DF, Thomas WH (1985). Computed tomography of the hindfoot. Arthritis Rheum.

