# Correction: Modified hindfoot alignment radiological evaluation and application in the assessment of flatfoot

**DOI:** 10.1186/s12891-023-06898-6

**Published:** 2023-09-29

**Authors:** Jing-Qi Liang, Yan Zhang, Liang Liu, Xiao-Dong Wen, Pei-Long Liu, Xin-Quan Yang, Xiao-Jun Liang, Hong-Mou Zhao

**Affiliations:** Foot and Ankle Surgery Department, Honghui Hospital of Xi’an Jiaotong University, Xi’an, China

**Correction:** ***BMC Musculoskelet Disord*** **24, 683 (2023)**

10.1186/s12891-023-06824-w

Following the publication of the original article [1], the authors noticed that the note for equal contributors “†Jing-Qi Liang and Yan Zhang contributed equally to this work.” did not appear in the proof.

The original article [1] has been updated.
